# The severity of initial acute kidney injury at admission of geriatric patients significantly correlates with subsequent in-hospital complications

**DOI:** 10.1038/srep13925

**Published:** 2015-09-10

**Authors:** Chia-Ter Chao, Hung-Bin Tsai, Chia-Yi Wu, Yu-Feng Lin, Nin-Chieh Hsu, Jin-Shing Chen, Kuan-Yu Hung

**Affiliations:** 1 Department of Medicine, National Taiwan University Hospital Jin-Shan branch, New Taipei City, Taiwan; 2 Department of Internal Medicine, National Taiwan University Hospital, Taipei, Taiwan; 3 Traumatology, National Taiwan University Hospital, Taipei, Taiwan; 4 Graduate Institute of Toxicology, National Taiwan University Hospital, Taipei, Taiwan; 5 The Department of Nursing, National Taiwan University, Taipei, Taiwan

## Abstract

Acute kidney injury (AKI) is associated with higher hospital mortality. However, the relationship between geriatric AKI and in-hospital complications is unclear. We prospectively enrolled elderly patients (≥65 years) from general medical wards of National Taiwan University Hospital, part of whom presented AKI at admission. We recorded subsequent in-hospital complications, including catastrophic events, incident gastrointestinal bleeding, hospital-associated infections, and new-onset electrolyte imbalances. Regression analyses were utilized to assess the associations between in-hospital complications and the initial AKI severity. A total of 163 elderly were recruited, with 39% presenting AKI (stage 1: 52%, stage 2: 23%, stage 3: 25%). The incidence of any in-hospital complication was significantly higher in the AKI group than in the non-AKI group (91% *vs.* 68%, *p *< 0.01). Multiple regression analyses indicated that elderly patients presenting with AKI had significantly higher risk of developing any complication (Odds ratio [OR] = 3.51, *p *= 0.01) and new-onset electrolyte imbalance (OR = 7.1, *p *< 0.01), and a trend toward more hospital-associated infections (OR = 1.99, *p *= 0.08). The risk of developing complications increased with higher AKI stage. In summary, our results indicate that initial AKI at admission in geriatric patients significantly increased the risk of in-hospital complications.

The elderly population is growing globally, and their healthcare needs will create tremendous financial burdens for developed and developing countries. The comorbidities that accompany aging increase the risk of subtle and frank organ dysfunction^1^. In particular, aging kidneys undergo functional decline and pathologic alterations, and this is accompanied by increasing sensitivity to nephrotoxins^2^,^3^. Population studies reported that the incidence of acute kidney injury (AKI) is 13 episodes per 1000 patient-years for patients aged 66 to 69 years, and is 47 episodes per 1000 patient-years for those older than 85 years^4^. This suggests a forthcoming worldwide increase of geriatric AKI, although few studies have examined this important topic.

AKI in the elderly is associated with prolonged hospitalization, increased risk of transfer to an intensive care unit (ICU), subsequent institutionalization, and increased short-term and long-term mortality^5^,^6^. In addition, advanced age appears to be a risk factor for poor renal recovery from AKI, because a higher proportion of elderly AKI patients develop chronic kidney disease (CKD) or end-stage renal disease (ESRD)^7^,^8^. A recent meta-analysis found that elderly patients (≥65 years) with AKI have a 28% higher risk of persistent disease than younger patients^7^. The majority of elderly AKI patients undergo functional decline, although their quality-of-life appears to remain stable or decrease only slightly^9^,^10^.

Previous studies of AKI in the elderly mostly examined short-term outcomes (in-hospital mortality) and long-term outcomes (renal prognosis, cardiovascular or all-cause mortality). However, the detrimental effects of AKI in geriatric patients might extend beyond survival or dependence on dialysis, because even mild renal insult in the elderly can have a negative impact^11^,^12^. Previous research suggests that more than half of hospitalized geriatric patients with AKI had stage I disease (defined by risk-injury-failure-loss-end stage [RIFLE] or acute kidney injury network [AKIN] staging)^11^,^12^. It is possible that milder degrees of AKI might also confer a higher risk for adverse events in hospitalized elderly (such as in-hospital complications), in addition to survival.

In the current study, we investigated the relationship of severity of AKI in geriatric in-patients with subsequent complications during their hospitalization.

## Materials and Methods

### Study design

This study protocol was approved by the National Taiwan University Hospital (NTUH) ethics committee (NO.201306089RINA), and all enrolled patients provided informed consent. The conduction of this study adhered to the Declaration of Helsinki. All elderly patients (≥65 years) who were admitted to the general medical wards of the NTUH between January 2014 and August 2014 were prospectively enrolled. Clinical data, including demographic profile (age, gender, body mass index [BMI]) and comorbidities were recorded at presentation. Charlson’s comorbidity index was utilized to describe the severity of comorbidities. Physical examination data (initial blood pressure, heart rate, respiratory rate, consciousness level) were also recorded. Admission diagnoses were categorized as cardiovascular disorders, airway and pulmonary diseases, hepatobiliary diseases, gastrointestinal diseases, renal and genitourinary disorders, sepsis from any organs/tissues or unknown origin, oncology (malignancy-related treatment or complications), or others. Initial laboratory test data were also collected.

AKI, characterized as an acute change in estimated glomerular filtration rate (eGFR), was defined according to the creatinine criteria of Kidney Disease Improving Global Outcomes (KDIGO) classification, based on the level of serum creatinine (sCr) on emergency room admission^13^. Stage 1 was defined by an increase of sCr by 50 to 100% within 7 days or to 0.3 mg/dL or more above baseline within 48 h; stage 2 was defined by an increase of sCr by 100 to 200% from baseline; and stage 3 was defined by a 200% or more increase of sCr, an increase to 4 mg/dL or more, or initiation of renal replacement therapy. Baseline sCr was determined from pre-admission data in all patients within three months. Serum creatinine was obtained from all participants at the day of admission.

### Outcome measures

The main outcome measure was development of any in-hospital complications, including a catastrophic event necessitating ICU transfer (myocardial infarction, acute respiratory failure, cardiopulmonary arrest with resuscitation, etc.), incident gastrointestinal bleeding, new-onset electrolyte imbalance, and any hospital-associated infection (HAI). An episode of incident gastrointestinal bleeding was monitored throughout hospitalization. We included macroscopic bleeding episodes and clinically suspicious bleeding episodes, with confirmation from positive stool occult blood assay. New-onset electrolyte imbalance was documented if any electrolyte in the electrolyte panel was normal at the emergency department and/or at out-patient department visits, but became abnormal after admission. The serum biochemistry panel and electrolyte profile (sodium, potassium, calcium, and phosphate) were routinely checked 1 or 2 times per week after admission, but serum magnesium was assayed if deemed necessary by the attending physician. Dysnatremia, dyskalemia, dyscalcemia, dysphosphatemia, and dysmagnesemia were each defined as the following: dysnatremia as hyponatremia (Na < 135 mmol/L) and/or hypernatremia (Na > 145 mmol/L), dyskalemia as hypokalemia (K < 3.5 mmol/L) and/or hyperkalemia (K > 5.0 mmol/L), dyscalcemia as hypocalcemia (calcium < 2.0 mmol/L) and/or hypercalcemia (calcium > 2.6 mmol/L), dysphosphatemia as hypophosphatemia (phosphate < 2.5 mg/dL) and/or hyperphosphatemia (phosphate > 5 mg/dL), and dysmagnesemia as hypomagnesemia (magnesium < 1.7 mg/dL) and/or hypermagnesemia (magnesium > 2.2 mg/dL). A HAI was coded according to infection-attributable symptoms and signs that occurred at least 48 h after admission, and positive microbiologic culture results^14^,^15^. The spectrum of HAI episodes in this study included pneumonia, urinary tract infection, cellulitis, *Clostridium difficile* colitis, and others.

### Statistical analysis

Statistical analyses were performed using SPSS 18.0 software (SPSS Inc., Chicago, IL, USA). Continuous variables are given as means ± standard deviations, and group comparisons were performed with an independent samples *t*-test or a Mann-Whitney *U*-test. Categorical variables are given as event numbers with percentages, and group comparisons were performed with a Chi-square test. We first categorized all patients according to the presence or absence of AKI, and compared clinical variables, comorbidities, vital signs, and diagnosis category at admission. Patients with AKI were further stratified by KDIGO stage, and within-group differences were analyzed with a one-way ANOVA. The effect of AKI on in-hospital complications was determined by multiple regression analyses that incorporated demographic profiles, comorbidities, admission vital signs and diagnoses, and variables involving AKI (with or without AKI, and AKI stages) into models, with stepwise variable selection. A two-sided *p*-value less than 0.05 was considered statistically significant.

## Results

### Characteristics of enrolled patients

A total of 163 elderly patients were prospectively enrolled during the 8-month study period, and 64 of these patients (39.3%) presented initially with AKI. Among these 64 patients, 33 (51.6%) had stage 1 AKI, 15 (23.4%) had stage 2 AKI, and 16 (25%) had stage 3 AKI. Only one of the stage 3 patients received acute dialysis. The origin of AKI could be attributed to acute tubular necrosis (resulting from sepsis or shock) (81.2%), cardiorenal syndrome (14.1%), dehydration (3.1%), and hepatorenal syndrome (1.6%).

Comparison of patients with and without AKI and of those with different stages of AKI indicated no significant differences in age, gender, or BMI (Table 1). Patients with AKI at presentation were significantly more likely to have chronic kidney disease (CKD) (*p *= 0.04), and CKD was more common in patients with more advanced AKI (*p *< 0.01). Among patients with different stages of AKI, there were no significant differences in the presence of diabetes mellitus (DM), vascular disease, pulmonary disorders, hemiplegia, or dementia/Parkinsonism. Overall, elderly patients with increasing severity of AKI had progressively more comorbidities, as demonstrated by higher Charlson’s comorbidity scores (p < 0.01).

On presentation, the overall mean systolic blood pressure (SBP) and diastolic blood pressure (DBP) were 134.9 mmHg and 74.7 mmHg, and patients with AKI had significantly lower SBP and DBP than patients without AKI (*p *< 0.01 for both comparisons). Patients with and without AKI had no significant differences in heart rate, respiratory rate, and category of diagnosis (Table 2). Patients with AKI had significantly lower platelet counts than those without AKI (*p *= 0.01), but similar leukocyte count and hemoglobin level. Patients with stage 3 AKI had significantly higher baseline serum creatinine than those with stage 1 and 2 AKI (*p *< 0.01), but there was no difference between patients with and without AKI.

### Clinical courses and outcomes

**Figure 1:**
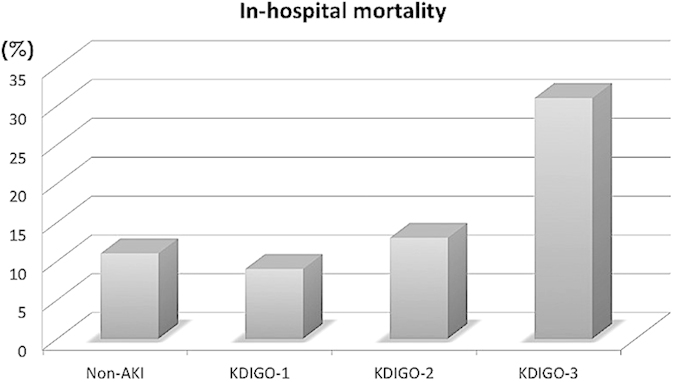
In-hospital mortality of geriatric patients without AKI and with stage 1, 2, and 3 AKI. Abbreviations: AKI, acute kidney injury; KDIGO, Kidney Disease Improving Global Outcomes.

The overall mean duration of hospitalization was 15.7 days, and there was a trend for longer hospitalization in patients with more advanced AKI. The overall mortality was 12.9%, as non-AKI group had 11% hospital mortality while AKI group on average had 16% hospital mortality. The in-hospital mortality increased significantly with AKI stage (9.1% for stage 1, 13.3% for stage 2, and 31.2% for stage 3; *p *= 0.04) (Fig. 1).

The overall rate of in-hospital complications was 77% (Table 3), and the most common complications were electrolyte imbalance (56%), gastrointestinal bleeding (50%), and HAI (25%). Catastrophic events necessitating ICU transfer were rare (3 patients; 2%). Two of them were transferred due to aspiration pneumonia with pulseless electrical activity, after receiving cardiopulmonary resuscitation. The third patient was transferred due to hospital-acquired pneumonia with septic shock. The incidence of any in-hospital complications was significantly higher in the AKI group than in the non-AKI group (*p *< 0.01) (Table 3). Among these complications, the incidence of HAI (*p *= 0.04) and new electrolyte imbalance (*p *< 0.01) were significantly higher in the AKI group than the non-AKI group, but the rate of incident gastrointestinal bleeding (*p *= 0.46) and catastrophic events (p = 0.85) were similar.

Dyskalemia was the most common electrolyte imbalance (43%), followed by dysnatremia (31%), dysmagnesemia (9%), dysphosphatemia (7%), and dyscalcemia (4%). The AKI group had significantly higher incidence of dysnatremia (*p *< 0.01) and dyskalemia (*p *= 0.02) than the non-AKI group. In addition, patients with more advanced AKI had significantly higher risk of dyscalcemia (*p *= 0.05) and dysphosphatemia (*p *= 0.02).

Next, we used multiple regression analyses to determine the significant predictors of in-hospital complications. The results show that AKI at presentation in elderly patients was associated with a significantly higher risk of any in-hospital complication (odds ratio [OR] = 3.51, 95% confidence interval [CI] = 1.32–9.3, *p *= 0.01) (Table 4). In addition, more advanced AKI was associated with higher risk of any in-hospital complication (stage 1: OR = 3.44, 95% CI = 1.11–10.7, *p *= 0.03; stage 2: OR = 6.39, 95% CI = 1.01–51.2, *p *= 0.05; stage 3: OR = 6.39, 95% CI = 1.01–51.3, *p *= 0.05). Furthermore, AKI at presentation was associated with increased risk of new electrolyte imbalance during hospitalization (OR = 7.1, 95% CI = 3.19–15.8, *p *< 0.01) and also with a trend toward increased risk of HAI (OR = 1.99, 95% CI = 0.92–4.29, *p *= 0.08). Similarly, more advanced AKI was associated with higher risk of new electrolyte imbalance during hospitalization (stage 1: OR = 5.37, 95% CI = 2.1–13.7, *p *< 0.05; stage 2: OR = 7.08, 95% CI = 1.8–27.9, *p *< 0.05; stage 3: OR = 39.9, 95% CI = 4.4–362.9, *p *< 0.01).

**Figure 2:**
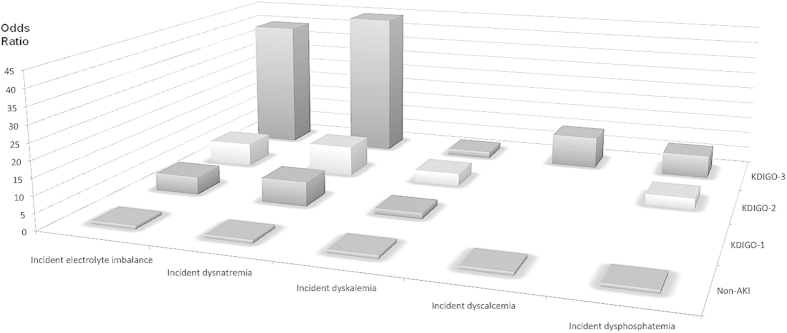
Risk of incident electrolyte imbalances in geriatric patients without AKI and with stage 1, 2, and 3 AKI. Abbreviations: AKI, acute kidney injury.

Finally, we subdivided the results of the regression analyses for the different types of electrolyte imbalances. Patients who had more severe AKI had higher risk of dysnatremia, dyskalemia, dyscalcemia, and dysphosphatemia, but there was no such effect for dysmagnesemia (Fig. 2). Relative to non-AKI patients, patients with stage 3 AKI had 44.6-fold increased risk of dysnatremia, 2-fold increased risk of dyskalemia, 9.9-fold increased risk of dyscalcemia, and 7-fold higher risk of dysphosphatemia.

Sensitivity analyses were performed to account for the effect of medications used before and after admission (including diuretics). The analysis results were essentially the unaltered. In addition, we tried to account for the effect of different definitions of baseline serum creatinine determination. Limiting analysis to those with baseline creatinine available within one month before AKI, we found that those with AKI were more likely to develop subsequent electrolyte imbalance (without vs. with, 47% vs. 79%, *p *= 0.01), and a trend toward higher likelihood of any in-hospital complications (without vs. with, 78% vs. 89%, *p *= 0.24). Elderly with more severe AKI also had significantly higher mortality (stage 1 vs. 2 vs. 3, 12% vs. 17% vs. 60%, *p *= 0.02), more likely to develop electrolyte imbalance (stage 1 vs. 2 vs. 3, 71% vs. 83% vs. 100%, *p *= 0.05), and hospital-associated infections (stage 1 vs. 2 vs. 3, 12% vs. 67% vs. 47%, *p *= 0.04), and a trend toward higher likelihood of any in-hospital complications (stage 1 vs. 2 vs. 3, 82% vs. 100% vs. 100%, *p *= 0.43). Another analysis excluding five patients with lower degree of creatinine elevation to over 4 mg/dL but categorized as stage 3 AKI showed essentially similar results; increasing AKI severity still showed significantly higher mortality (stage 1 vs. 2 vs. 3, 9% vs. 13% vs. 50%, *p *< 0.01), more any in-hospital complications (stage 1 vs. 2 vs. 3, 88% vs. 93% vs. 100%, *p *= 0.01), and more electrolyte imbalance (stage 1 vs. 2 vs. 3, 76% vs. 80% vs. 100%, *p *= 0.01). Multiple regression analysis showed that presence of AKI increased the risk of any in-hospital complications (OR = 4.83, 95% CI = 1.6–14.4, *p *< 0.01) and electrolyte imbalance (OR = 8.43, 95% CI = 3.6–19.6, *p *< 0.01). Analysis excluding patients with pre-existing CKD also showed that those with AKI were more likely to develop any in-hospital complications (without vs. with, 69% vs. 91%, *p *< 0.01) and electrolyte imbalance (without vs. with, 43% vs. 79%, *p *< 0.01). Patients with increasing AKI severity were more likely to develop any in-hospital complications (stage 1 vs. 2 vs. 3, 89% vs. 92% vs. 100%, *p *= 0.05) and electrolyte imbalance (stage 1 vs. 2 vs. 3, 78% vs. 85% vs. 100%, *p *= 0.02). Finally, presence of AKI was associated with a significantly higher risk of developing any in-hospital complications (OR = 4.51, 95% CI = 1.5–14, *p *< 0.01) and electrolyte imbalance (OR = 5, 9% CI = 2.1–11.8, *p *< 0.01).

## Discussion

The results of this study indicate that elderly patients with initial AKI at admission had greater risk of developing subsequent in-hospital complications, including new infections and electrolyte imbalances. The risk of developing subsequent complications increased with the initial severity of AKI. Sub-analyses indicated that more severe initial AKI significantly increased the risk of new-onset electrolyte imbalances (especially sodium, potassium, calcium, and phosphate) and of HAIs during hospitalization. These findings are the first of their kind, and suggest that AKI has an important impact on hospital complications in addition to its established effect on in-hospital mortality.

The incidence of AKI in the elderly is typically higher than in the general population because the elderly are more likely to have renal structural decline and multiple comorbidities^16^,^17^. Previous studies reported the incidence of AKI among geriatric in-patients as 22 to 40%, with most patients having stage 1 disease^12^,^18^,^19^,^20^. We found that 39.3% of geriatric patients had AKI, in agreement with the literature^21^. Our findings are at the higher end likely due to the advanced age of our patients. The in-hospital mortally of our geriatric AKI patients (16%) was also similar to other studies (16–20%)^11^,^12^.

Hospitalized patients have a higher risk of developing in-hospital complications, but the exact incidence varies according to complication definitions and the reasons for hospitalization. Most studies addressed post-operative complications. In the elderly, the incidence of in-hospital complications is expectedly higher because of organ decline, decreased physiologic reserves, frailty, and the higher prevalence of disability and functional impairment^22^. Indeed, several reports indicated that elderly patients have a 20 to 30% greater incidence of hospital complications after surgery^23^,^24^. This number would be even greater if the complication definitions extend beyond those related to surgery. A systematic review suggested that elderly institutional residents had a 38 to 80% incidence of in-hospital complications^25^. In our cohort, we identified geriatric complications including incident gastrointestinal bleeding and infections, based on modifications of past studies. Our estimates (77%) were then compatible with other reports in geriatric patients.

Determining baseline sCr could be difficult for studies of AKI, and is a highly debated issue. In many cases, there is no sCr available within seven days prior to admission, a requirement for accurately defining AKI. Several methods have been proposed to deal with this conundrum; first, a back-calculation of baseline creatinine using Modification Diet in Renal Disease (MDRD) equation, assuming a baseline eGFR of 75 ml/min/1.73 m^2^, could be considered^26^. However, such assumption may be at risk for over-estimating AKI incidence especially for patients with pre-existing renal diseases, which would be common in elderly patients^27^. In addition, others suggested that nadir creatinine values within the index hospitalization could be used to estimate baseline sCr. Since elderly patients are vulnerable to sepsis and fluid accumulation, this phenomenon might compromise the capacity of nadir creatinine values to estimate baseline sCr^28^. Finally, a pre-morbid creatinine values measured longer than seven days before events could be useful. We opted to use pre-morbid creatinine measurements within ninety days before AKI, in order to enhance the accuracy of capturing AKI in this elderly cohort. Multiple studies have validated the utility of this approach. Siew *et al.* found that sCr measured between 7 days and 1 year before admission exhibited a very high degree of agreement with reference baseline creatinine values^29^. The recently released international consensus on AKI diagnosis in cirrhotic patients also endorsed this approach^30^.

The presentation of lower initial SBP and DBP in elderly patients with AKI is interesting (Table 2). We suggest that this manifestation might result from the causes of AKI. In our cohort, 81.2% of the elderly AKI were related to sepsis or shock related acute tubular necrosis, and it is expected that patients with AKI might exhibit lower blood pressure than those without. In addition, baseline serum creatinine did not differ between elderly with and without AKI (Table 2). We propose that sarcopenia, which is commonly observed in elderly patients, could be one potential explanation. Sarcopenia is frequently associated with fluctuated sCr levels, and could interfere with the ability of serum creatinine to act as surrogate for renal function.

Electrolyte imbalance is an under-recognized hospital complication, but is relatively common among elderly in-patients, as our study found (40%)^31^. Through interfering with tubular functions, AKI disturbs the physiologic regulation of electrolyte homeostasis, which could be more prominent in the elderly. Previous studies have consistently reported that electrolyte imbalances, including dysnatremia and dyskalemia, are associated with worse in-hospital outcomes^31^,^32^. Furthermore, the adverse impact of dysnatremia on clinical outcomes might be independent of the presence of initial AKI. Even mild hyponatremia (130–135 meq/L) or mild hypernatremia (145–150 meq/L) predicts significantly higher hospital mortality, while correction of dysnatremia is associated with improved survival^33^,^34^. Through quantifying the fold of risk elevation for dysnatremia, and other electrolytes, our findings could provide the rationale for devising individualized approaches to correct different types of dyselectrolytemia.

It is widely recognized that AKI is associated with prolonged hospitalization and poorer outcomes, but few studies have examined the association between AKI and in-hospital complications. Available reports often consider AKI as a type of hospital complication rather than a preceding or precipitating event. A large-scale study indicated that patients with AKI had a 1.9-fold higher risk of gastrointestinal bleeding during hospitalization^35^, and another study reported that episodes of post-operative AKI increase the risk of nosocomial infections^36^. In the present study, we found that initial AKI raises risk of in-hospital complications in the elderly, particularly dyselectrolytemia (Table 4). In support of this finding, these risks increased incrementally with higher AKI stage. These relationships potentially shed light on the elusive relationship between initial AKI early in the hospitalization course and subsequent adverse in-hospital events. However, we did not detect statistically significant associations between initial AKI and subsequent incident GI bleeding or HAIs (Table 4). These negative findings could partially result from an inadequate case or event number, the definitions of GI bleeding/HAIs we used in this study, or the spectrum of elderly patients we enrolled. Nonetheless, our finding in elderly patients with AKI might still set the stage for future research in the elderly, who are more vulnerable to complications.

Our study has its strength and weakness. It is among the few reports to evaluate the risk of in-hospital complications due to AKI in elderly patients admitted for medical causes. A significantly elevated risk of overall in-hospital complications among elderly AKI patients of different severity, with dose-responsiveness, was detected, serving as an explanation for the mechanisms between AKI and its negative impact on short-term mortality. The negative effect of AKI varies according to types of electrolytes, and quantification of the risk for different electrolyte types might influence the intensity of care (frequency of follow up, medications used for correction, etc.) for elderly AKI patients. Furthermore, a pre-specified protocol directed at monitoring and also treating these in-hospital complications might carry the potential for improving outcomes of elderly patients with AKI. However, the limitation lies in its focus on geriatric patients, so that our findings might not be readily applicable to AKI patients in general. Our definition of in-hospital complications might not have been broad enough, and several complications were of lower frequency. Finally, the findings of this study may not be applicable outside of clinical settings.

## Conclusion

In this study, we found that the presence of AKI at admission of geriatric patients is associated with higher risk for in-hospital complications, specifically hospital-associated infections and electrolyte imbalances. Moreover, the risk of complications was greater for patients with more severe AKI. Further studies that incorporate different definitions of complications and that examine larger populations are needed to confirm our findings.

## Additional Information

**How to cite this article**: Chao, C.-T. *et al.* The severity of initial acute kidney injury at admission of geriatric patients significantly correlates with subsequent in-hospital complications. *Sci. Rep.* **5**, 13925; doi: 10.1038/srep13925 (2015).

## Figures and Tables

**Table 1:** Baseline characteristics of elderly patients (≥65 years) with and without acute kidney injury (n = 163).

Characteristic	Total	Without AKI	With AKI			p1 value	p2 value
			Stage 1	Stage 2	Stage 3		
Age (years)	80.3 ± 8.1	80.8 ± 8.1	79.9 ± 7.8	78.2 ± 8.1	79.5 ± 9.1	0.26	0.8
Gender (male %)	80 (49)	44 (45)	18 (55)	8 (53)	10 (63)	0.12	0.85
BMI (kg/m^2^)	22.2 ± 5.2	21.8 ± 6.1	23 ± 3.6	23.6 ± 3.5	21.8 ± 2.4	0.36	0.73
DM (%)	65 (40)	33 (34)	16 (48)	7 (47)	9 (56)	0.07	0.85
Hypertension (%)	92 (56)	59 (60)	19 (58)	2 (13)	12 (75)	0.25	<0.01
CAD (%)	12 (7)	8 (8)	2 (6)	0 (0)	2 (13)	0.7	0.37
LC (%)	9 (6)	3 (3)	3 (9)	1 (7)	2 (13)	0.08	0.86
AMI (%)	1 (1)	0 (0)	0 (0)	0 (0)	1 (6)	0.21	0.23
Heart failure (%)	30 (18)	15 (15)	7 (21)	3 (20)	5 (31)	0.16	0.7
PAOD (%)	12 (7)	9 (9)	2 (6)	0 (0)	1 (6)	0.32	0.63
COPD (%)	18 (11)	10 (10)	6 (18)	1 (7)	1 (6)	0.6	0.38
CKD (%)	40 (25)	19 (19)	6 (18)	3 (20)	12 (75)	0.04	<0.01
RD (%)	4 (2)	1 (1)	3 (9)	0 (0)	0 (0)	0.13	0.24
Malignancy (%)	41 (25)	21 (21)	6 (18)	9 (60)	5 (31)	0.13	0.01
GI ulcer (%)	18 (11)	10 (10)	3 (9)	5 (33)	0 (0)	0.6	0.01
Hemiplegia (%)	5 (3)	4 (4)	1 (3)	0 (0)	0 (0)	0.39	0.63
Dementia/PD (%)	22 (13)	14 (14)	6 (18)	2 (13)	0 (0)	0.81	0.2
Charlson’s index (with age)	7.7 ± 2.4	7.4 ± 2.3	7.5 ± 2.5	8.8 ± 1.8	8.9 ± 2.4	0.03	0.02
Charlson’s index (without age)	3.1 ± 2.3	2.7 ± 2.3	2.9 ± 2.3	4.4 ± 2	4.4 ± 2.4	<0.01	<0.01

Data are expressed as mean ± standard deviation for continuous variables, and n (percentage) for categorical variables.

*p1*: Without AKI *vs.* With AKI, by independent Student’s *t*-test.

*p2*: KDIGO stage 1, stage 2, and stage 3, by one way ANOVA.

Abbreviations: AKI, acute kidney injury; AMI, acute myocardial infarction; BMI, body mass index; CAD, coronary artery disease; CKD, chronic kidney disease; COPD, chronic obstructive pulmonary disease; DM, diabetic mellitus; GI, gastrointestinal; LC, liver cirrhosis; PAOD, peripheral artery occlusive disease; PD, Parkinson’s Disease; RD, rheumatological disorder.

**Table 2:** Clinical data of elderly patients with and without acute kidney injury at presentation and during the course of hospitalization.

Clinical presentation	Total	Without AKI	With AKI			p1 value	p2 value	
			Stage 1	Stage 2	Stage 3			
SBP (mmHg)	134.9 ± 37.1	144.7 ± 36.2	128.6 ± 37.5	106.6 ± 30.2	113 ± 20	<0.01	0.08	
DBP (mmHg)	74.7 ± 20.3	80 ± 21.2	67.7 ± 15.2	69.1 ± 18.1	61.3 ± 14.7	<0.01	0.36	
HR (/min)	95.9 ± 21	97.4 ± 18.8	93.8 ± 25.1	99.6 ± 23.4	87.4 ± 21.2	0.21	0.41	
RR (/min)	20 ± 3.1	20.1 ± 3.1	19.7 ± 2.2	20.7 ± 5.1	19.5 ± 2.6	0.7	0.69	
GCS score	12.5 ± 3.1	12.5 ± 3.2	12.3 ± 3.1	12.1 ± 2.8	12.9 ± 2.8	0.97	0.91	
Main diagnosis at admission						0.44	0.07	
Cardio-vascular disorders (%)	7 (4)	4 (4)	3 (9)	0 (0)	0 (0)			
Pulmonary disorders (%)	73 (45)	47 (48)	17 (52)	4 (27)	5 (31)			
Hepatobiliary disorders (%)	11 (7)	6 (6)	3 (9)	0 (0)	2 (13)			
Gastrointestinal disorders (%)	15 (9)	6 (6)	3 (9)	3 (20)	3 (19)			
Renal disorders (%)	18 (11)	5 (5)	5 (15)	5 (33)	3 (19)			
Sepsis of other etiology (%)	14 (9)	12 (12)	0 (0)	1 (7)	1 (6)			
Oncology disorders (%)	11 (7)	7 (7)	1 (3)	2 (13)	1 (6)			
Miscellaneous illness (%)	14 (9)	12 (12)	1 (3)	0 (0)	1 (6)			
Initial laboratory data								
White blood cells (K/μL)	12.2 ± 6.2	11.6 ± 5.1	12.1 ± 7.3	15.3 ± 6.3	13.3 ± 9.1	0.18	0.16	
Hemoglobin (g/dL)	11.6 ± 8.4	12.6 ± 10.4	12.6 ± 10.5	10 ± 2.5	11.7 ± 2.7	0.06	0.23	
Platelet (K/μL)	225 ± 104	242 ± 104	198 ± 105	215 ± 106	187 ± 82	0.01	0.09	
Baseline Creatinine (mg/dL)	1.4 ± 1.4	1.3 ± 1.5	1.1 ± 0.5	0.9 ± 0.4	2.8 ± 1.7	0.33	<0.01	

p1 value: Without AKI *vs.* With AKI, by independent Student’s t-test.

p2 value: Between KDIGO stage 1, stage 2, and stage 3 AKI, by one way ANOVA.

Abbreviations: AKI, acute kidney injury; DBP, diastolic blood pressure; GCS, Glascow coma scale; HR, heart rate; RR, respiratory rate; SBP, systolic blood pressure.

**Table 3:** In-hospital complications of elderly patients with and without acute kidney injury.

Complications	Total	Without AKI	With AKI				p1 value	p2 value
			Total	Stage 1	Stage 2	Stage 3		
Any complication (%)	125 (77)	67 (68)	58 (91)	29 (88)	14 (93)	15 (94)	<0.01	0.75
Catastrophic events with ICU transfer	3 (2)	2 (1)	1 (2)	1 (3)	0 (0)	0 (0)	0.85	0.84
Hospital-acquired Infections (%)	40 (25)	19 (19)	21 (33)	11 (33)	6 (40)	4 (25)	0.04	0.68
Incident GI bleeding (%)	81 (50)	52 (53)	29 (45)	15 (45)	7 (47)	7 (44)	0.46	0.99
Incident electrolye imbalance (%)	91 (56)	39 (39)	52 (81)	25 (76)	12 (80)	15 (94)	<0.01	0.33
dysnatremia (%)	51 (31)	16 (16)	35 (55)	17 (52)	6 (40)	12 (75)	<0.01	0.13
dyskalemia (%)	70 (43)	35 (35)	35 (55)	16 (48)	10 (67)	9 (56)	0.02	0.51
dyscalcemia (%)	6 (4)	4 (4)	2 (3)	0 (0)	0 (0)	2 (13)	0.79	0.05
dysphosphatemia (%)	11 (7)	5 (5)	6 (9)	0 (0)	2 (13)	4 (25)	0.27	0.02
dysmagnesemia (%)	14 (9)	7 (7)	7 (11)	4 (12)	3 (20)	0 (0)	0.37	0.2
Hospitalization duration (days)	15.7 ± 16.4	14.1 ± 11.2	18.2 ± 22.1	14.7 ± 13.7	20 ± 13.6	23.5 ± 37.3	0.11	0.4

*p1* value: Without AKI vs. With AKI, by independent Student’s t-test.

*p2* value: Between KDIGO stage 1, stage 2, and stage 3 AKI, by one way ANOVA.

Abbreviations: AKI, acute kidney injury; GI, gastrointestinal; ICU, intensive care unit.

**Table 4:** Multiple regression analyses of elderly patients with and without acute kidney injury, with in-hospital complications as the dependent variables.

Results	Odds ratio	95% Confidence Interval	p value
Model 1a – All in-hospital complications			
AKI at presentation	3.51	1.32–9.3	0.01
SBP at presentation	0.99	0.98–1.0	0.08
Model 1b – All in-hospital complications			
AKI KDIGO stage 1	3.44	1.11–10.7	0.03
AKI KDIGO stage 2	6.39	1.01–51.2	0.05
AKI KDIGO stage 3	6.39	1.01–51.3	0.05
Model 2 – New-onset hospital-associated infections			
DM as comorbidity	2.35	1.09–5.07	0.03
AKI at presentation	1.99	0.92–4.29	0.08
Model 3a – Incident electrolyte imbalances			
AKI at presentation	7.1	3.19–15.8	<0.01
Model 3b – Incident electrolyte imbalances			
AKI KDIGO stage 1	5.37	2.1–13.7	<0.01
AKI KDIGO stage 2	7.08	1.8–27.9	<0.01
AKI KDIGO stage 3	39.9	4.4–362.9	<0.01

Models 1 and 3 include variables from demographic data, all comorbidities, Charlson comorbidity index, vital signs, admission diagnoses, and AKI (with *vs.* without for 1a and 3a) or AKI (divided into tertiles for 1b and 3b).

Model 2 includes variables from demographic data, all comorbidities, Charlson comorbidity index, vital signs, admission diagnoses, and AKI (with *vs.* without).

Abbreviations: AKI, acute kidney injury; DM, diabetes mellitus; KDIGO, Kidney Disease Initiative Global Outcome; SBP, systolic blood pressure.
